# Innovative Approaches to Poultry Processing Wastewater Treatment: The Stainless Steel Ultrafiltration Membrane as a Viable Option

**DOI:** 10.3390/membranes13110880

**Published:** 2023-11-11

**Authors:** Saubana Olorunsola Dada, Chidambaram Thamariselvan, Mahmood Jebur, Sumith Ranil Wickramasinghe

**Affiliations:** 1 Ralph E. Martin Department of Chemical Engineering, University of Arkansas, Fayetteville, AR 72701, USA; sodada@uark.edu (S.O.D.); mgjebur@tu.edu.iq (M.J.); 2 Interdisciplinary Centre for Energy Research, Indian Institute of Science, Bengaluru 560012, India

**Keywords:** poultry processing wastewater, stainless steel membrane, water treatment, critical flux, flux decline, chemical oxygen demand

## Abstract

In pursuit of sustainability, we explored replacing conventional dissolved air floatation (DAF) in poultry processing wastewater (PPW) treatment with a precisely tuned 0.02 µm stainless-steel ultrafiltration (SSUF) membrane. SSUF is a robust, homogenously porous membrane with strong chemical resistance, ease of cleaning, and exceptional resistance to organic fouling. Unlike polymeric membranes, it can be regenerated multiple times, making it a cost-effective choice due to its compatibility with harsh chemical cleaning. The PPW used for the study was untreated wastewater from all processing units and post-initial screening. Our study revealed the SSUF membrane’s exceptional efficiency at eliminating contaminants. It achieved an impressive removal rate of up to 99.9% for total suspended solids (TSS), oil, grease, *E. coli*, and coliform. Additionally, it displayed a notable reduction in chemical oxygen demand (COD), biochemical oxygen demand (BOD), and total Kjeldahl nitrogen (TKN), up to 90%, 76%, and 76%, respectively. Our investigation further emphasized the SSUF membrane’s ability in pathogen removal, affirming its capacity to effectively eradicate up to 99.99% of *E. coli* and coliform. The measured critical flux of the membrane was 48 Lm^−2^h^−1^ at 38 kPa pressure and 1.90 m/s cross-flow velocity. In summary, our study highlights the considerable potential of the SSUF membrane. Its robust performance treating PPW offers a promising avenue for reducing its environmental impact and advocating for sustainable wastewater management practices.

## 1. Introduction

Meat consumption in the United States (US) is continuously growing. Roughly three times the world’s average meat is consumed in the US. While red meat consumption in the US has remained oscillating between the same figure for the past 100 years, the demand for poultry has continued to rise steadily. Based on the current trend, the amount of poultry consumed in the US is expected to surpass red meat in a few years [1,2]. Recently, more than 9 billion poultry are killed in the US annually [3,4]. The steps involved in converting a bird to meat include slaughtering, scalding, picking, eviscerating, washing, chilling, weighing, packaging, and transportation [5]. All of these steps require water [6], with large percentage being used for eviscerating, bird washing, cutting, and chilling [7,8]. About 26.5 L of water is required for bird processing. Mathematically, over 230 billion liters of water is being used to process birds annually, without accounting for water used in raising the birds. The large volume of water used for processing poultry necessitates the need for sustainable usage. Recycling and reusing wastewater not only conserves water resources, but also enhances water quality [9,10,11].

Poultry processing wastewater (PPW) is highly contaminated, primarily with blood and rejected poultry waste known as offal. Kiepper et al. [6] describe offal as the inedible parts of the poultry, such as the feathers, head, intestines, and other discarded parts. The concentration of contaminants in PPW can fluctuate from day to day across various processing units [12]. The specific composition of PPW is typically determined by measuring various parameters influenced by factors like the duration of blood draining and the presence of offal [5,7,13]. Numerous studies have explored the characterization of PPW from different sections of poultry processing plants, including the chilling section [13]; washing section [5]; and sections responsible for de-feathering, eviscerating, and cooling [14]. Interestingly, it has been observed that the combined wastewater has the highest impurity content [15]. Understanding the particle size distribution in PPW is essential for appropriate membrane selection. Previous studies have reported varying particle sizes in PPW. While the average particle size within PPW has been reported to be approximately 0.14 µm, the chilling section PPW has an average particle size of 0.084 µm and the washer section’s average particle size is 0.375 µm, which is almost five times larger [5,13,15].

**Figure 1:**
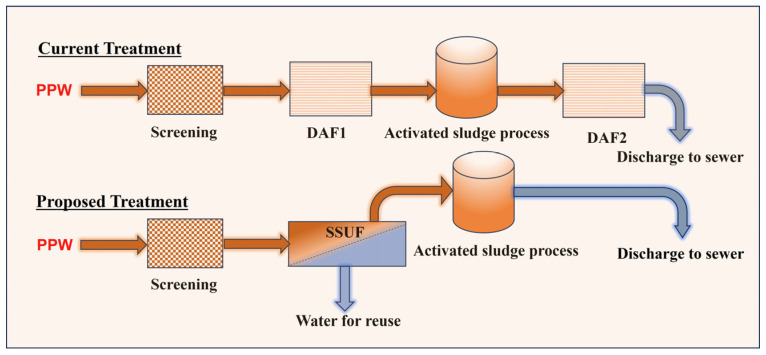
Schematic diagram of the current PPW treatment method and the anticipated method.

Like other industries, poultry processors must treat their wastewater, and the treated water must meet some standards before being discharged to sewers or the environment. In the US, dissolved air floatation (DAF) is a widely used method for treating slaughterhouse wastewater (including PPW). Figure 1 below illustrates the current treatment process for PPW in the processing plant and the proposed treatment method to be investigated in this study for comparison.

DAF has certain disadvantages, including the use of toxic chemicals and the requirement for large footprints. By-product recovery is also challenging with DAF due to content destabilization [10]. Studies have been completed on other potential techniques for treating PPW, e.g., sulphuric acid (H_2_SO_4_) precipitation followed by algae cultivation [15], as well as the combination of a screening system, equalization tank, DAF system, and up-flow anaerobic sludge blanket reactors [16]. Another technique explored for PPW treatment is membrane processes, such as ultrafiltration (UF). UF is a low-pressure driven technique with a pore size ranging from 0.001 to 0.1 µm. Different configurations of UF have been explored, including integrating UF systems with other techniques. UF performed remarkably in removing contaminants and microbes when PPW was treated with a standalone UF or UF-coupled systems for various purposes. Like other membrane operations, fouling is the main challenge with UF, and regeneration is needed after every usage [5,11,13,14,17,18,19,20]. The use of UF in the treatment of PPW has been a subject of research for several years. Shih et al. pioneered this approach by applying UF membranes to treat PPW and recover its valuable nutrients. Their work, dating back to the 1980s, highlighted the economic benefits and the potential to achieve dischargeable water quality [21]. While polymeric membranes are commonly employed, they often exhibit inherent hydrophobic properties, leading to significant fouling issues and a limited lifespan. Moreover, most polymeric membranes lack one of the critical attributes, such as chemical/thermal stability, pH resistance, or mechanical strength [22]. In contrast with polymeric membranes, ceramic membranes offer enhanced chemical and mechanical strength. However, they remain susceptible to fouling, especially biofouling, and suffer from high manufacturing costs [23,24]. Some researchers have explored the promise of metallic membranes, particularly stainless-steel membranes, for sewage treatment [25] and for the clarification of limed sugarcane juice [26]. Zhang et al. conducted research demonstrating that stainless steel membrane pores are exceptionally homogenous and effectively remove organic contaminants in sewage treatment with a stainless-steel membrane bioreactor. They also concluded that regular backwashing procedures could mitigate membrane fouling and extend the membrane’s lifespan, affirming the stainless-steel membrane’s effectiveness for treating PPW [25]. Using stainless-steel ultrafiltration (SSUF) will potentially eliminate many process units, thereby intensifying the process unit. Despite its advantages, SSUF has some limitations. It cannot be regenerated with some chemicals, such as hydrochloric acid (HCl), because they are reactive. Also, SSUF could be susceptible to corrosion.

Molecular adsorption, cake formation, and pore plugging are the main causes of membrane fouling, thereby reducing the membrane’s performance. It has been proposed to operate the membrane below critical flux (i.e., run below critical pressure). Critical flux is defined as the flux below which no irreversible membrane fouling depends on the hydrodynamics. Some studies have been conducted on critical flux measurement. The flux-step method, which entails increasing the flux and measuring the corresponding transmembrane pressure (*TMP*), has been used in several studies to determine the critical flux [27,28,29,30,31,32].

In this study, we investigated the use of SSUF membranes for treating PPW and the SSUF effective regeneration method, and we explored the feasibility of using SSUF as an alternative efficient method for the commercial DAF system in the poultry-produced water treatment industry. Also, we determined the SSUF critical flux. We used combined PPW taken after the screening process for this study as well as PPW with no pretreatment. We measured the performance in terms of the water recovery potential and the removal efficiency of biochemical oxygen demand (BOD), soluble BOD (sBOD), chemical oxygen demand (COD), total soluble solids (TSS), oil and grease, and total Kjeldahl nitrogen (TKN). To our knowledge, no work has been conducted on using SSUF to treat PPW. Short-term and long-term studies were performed to understand the membrane’s performance for treating PPW.

## 2. Materials and Methods

### 2.1. Materials

PPW was obtained from Tyson Foods Inc. (Springdale, AR, USA). Combined wastewater from all units was collected at the entrance to the first DAF. Initially, we stored the PPW in a well-sealed container, which was further protected with a plastic bag. However, after a few days, we observed alterations in the properties of PPW. These changes included a shift in color, a noticeable increase in unpleasant odor due to bacterial growth, and the formation of agglomerated particles. Industrially, PPW is treated immediately, and using the changed PPW could lead to misleading results. As a result of these observed changes, same-day PPW was used for all of the studies. The materials used for the experiments were a 2.5 gallon (9.5 L) NSF-certified feed tank from Ace Roto-Mold (Hospers, IA, USA), an analytical scale from Mettler Toledo (Columbus, OH, USA), a pumping system with a motor created by Regal Rexnord (Beloit, WI, USA) and a head created by Wagner Engineering (Minneapolis, MN, USA), a control system created by TECO Westinghouse (Round Rock, TX, USA), and a commercially available SSUF module from Scepter^®^ a registered trademark owned and operated by Graver Technologies (Glasgow, DE, USA). Table 1 shows the membrane specifications.

### 2.2. Experimental Methods

**Figure 2:**
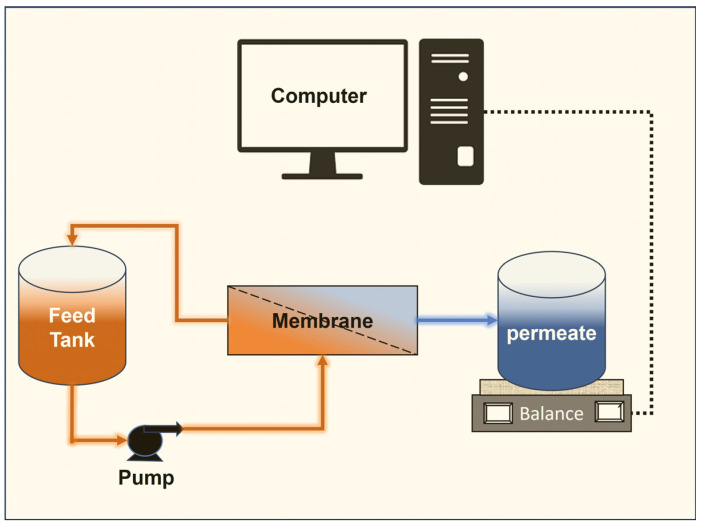
Schematic diagram of the lab scale experimental setup.

As illustrated in Figure 2, the setup consisted of a pumping system, feed tank, membrane, weighing scale, and desktop computer. To monitor the PPW temperature during the experiment, a temperature sensor was attached to the feed tank. Initially, the system was flushed using deionized (DI) water to make sure there were no contaminants or cleaning agents that could contaminate the PPW. Then, 5.7 L of thoroughly mixed PPW was added to the NSF-certified feed tank following DI rinsing. We began the investigation by making sure the membrane exit valve was entirely closed, and the input and recirculation valves were fully opened after the tank had been filled. After starting the pump, the pressure gauge reading fluctuated at first. The permeate side of the system was closed for a brief period of time while the system ran until the pressure reading stabilized.

Once the reading in the pressure gauge stabilized, the pump was adjusted until the desired feed flow rate of 0.85 GPM (0.000063 m^3^/s) was achieved. The feed flow rate corresponded to the pump speed of 50 revolutions per minute (rpm). The maximum allowable speed of the pump was 60 rpm, and we decided to operate the pump with a 10 rpm allowance. The permeate side valve was then completely opened, and the control valve at the membrane outflow was set to obtain a specified *TMP*. The *TMP* was computed as follows:

(1) $$TMP=\frac{\left(Pf+Pr\right)}{2}-Pp$$

where *Pf* is feed pressure, *Pr* is retentate pressure, and *Pp* is permeate pressure.

The permeate pressure was zero for this filtration experiment, and the equation above became the following:

(2) $$TMP=\frac{\left(Pf+Pr\right)}{2}$$

Every 15 min, permeate mass was collected and measured with a weighing scale attached to a computer. The temperature data from the feed tank sensor were recorded, and the flux was normalized to 25 °C using the viscosity at the noted temperature. To normalize the flux, we used the water viscosity at the recorded temperature. The viscosity was divided by the viscosity of water at 25 °C, and the ratio was multiplied by the flux recorded at that specific time. The *TMP* was constant for each experiment. Initially, it was decided that at least 10% of the PPW needed to be recovered for a complete cycle. For the first set of experiments, 10% of the fed PPW was collected after 6 h, and subsequent studies were created to last for 6 h. Experiments were performed at 276 kPa, 483 kPa, and 758 kPa *TMP* for 6 and 10 h.

### 2.3. Critical Flux Experiments

The identical experimental arrangement (Figure 2) was employed to ascertain the critical flux of the membrane. To control the permeate side pressure, a pressure gauge was put in place on the membrane’s permeate side. The feed tank was filled with 3.8 L of PPW only after the permeate side valve had been completely closed. After turning on the pump, the flow was adjusted to the required rate. After a few minutes of system stabilization, the permeate valve was somewhat opened. The feed, retentate, and permeate pressure readings were observed, and adjustments were made until the target *TMP* was reached. For 30 min, the flux was recorded. The flux was monitored for 30 min after the permeate pressure gauge was adjusted to raise the *TMP*. The *TMP* was switched around in order to repeat the experiment. Later on, the experimental time was also adjusted appropriately. In the previous study, the flux was periodically measured while *TMP* was gradually increased over time. Instead of using the flux-steps method by Yuliwati et al. [33], we used *TMP*-steps to calculate the critical flux values. In their work, they gradually increased the flux until the *TMP* did not change; but here, we increased the *TMP* gradually and noticed at what point the flux response to *TMP* change was not linear. The *TMP*-steps study was achieved by starting the study with a 10 min *TMP* stepping. The study started at zero *TMP*, and after every 10 min, the *TMP* was increased by 34 kPa. After the *TMP* increment, the flux was expected to increase. The procedure was conducted for 2 h. This step was repeated until the flux stopped responding to the pressure change, and critical flux was achieved.

### 2.4. Analytical Methods

A laser diffraction particle size analyzer (Beckman Coulter, LS 13 320, Brea, CA, USA) was used to measure the particle distribution in PPW prior to each experiment. The Tyson Food Rivers Valley Regional Laboratory (Scranton, AR, USA) then performed TSS, COD, BOD, pH, oil and grease, sBOD, and TKN characterizations on the PPW and permeate. The outcome served as a gauge for SSUF’s removal effectiveness. The total coliform count and *E. coli* count in the permeate and PPW were also measured at the Arkansas Water Resources Center (Fayetteville, AR, USA). The removal efficiency was determined using the following formula.

(3) $$Efficiency=\frac{Cppw-Ctreated}{Cppw}\times 100$$

The wastewater and permeate BOD were both examined using the 5-day BOD test. The outcome was used to calculate SSUF’s removal efficiency. The Arkansas Water Resources Center (Fayetteville, AR, USA) performed the coli count and total coliform count in the PPW and permeate. Utilizing the IDEXX Colilert 24–97 Well Tray (APHA 9223, B standard), pathogen removal was verified. Equation (3) was used to calculate the removal efficiency.

### 2.5. Membrane Regeneration

The membrane underwent regeneration after each experiment and we continued to use the same membrane for all experiments reported in this study. As the use of SSUF membranes for treating PPW was a new approach, we tested regeneration methods typically used for cleaning other types of membranes on SSUF membranes. To regenerate the membrane, it was initially filled with a solution containing Protease A at a concentration of 0.03 mg/mL and sodium dodecyl sulfate (SDS) at a concentration of 0.003 g/mL. The solution was heated to 38 °C and incubated for 24 h [34]. Subsequently, the membrane was thoroughly rinsed with deionized (DI) water. Next, we circulated a 1M sodium hydroxide (NaOH) solution mixed with 3 g of SDS in 1000 mL of water. After heating the solution to 60 °C, it was passed through the membrane for an hour, with the permeate side fully closed. After that, the membrane was rinsed with DI water for 5 min. The last step was to pass a 70 to 80 °C nitric acid (HNO_3_) solution (1% *v*/*v*) through the membrane for an hour. The membrane was then rinsed with DI water again [35]. After cleaning, we checked the SSUF membrane’s cleanliness by measuring the DI flux.

## 3. Results

### 3.1. PPW Characterization

**Figure 3:**
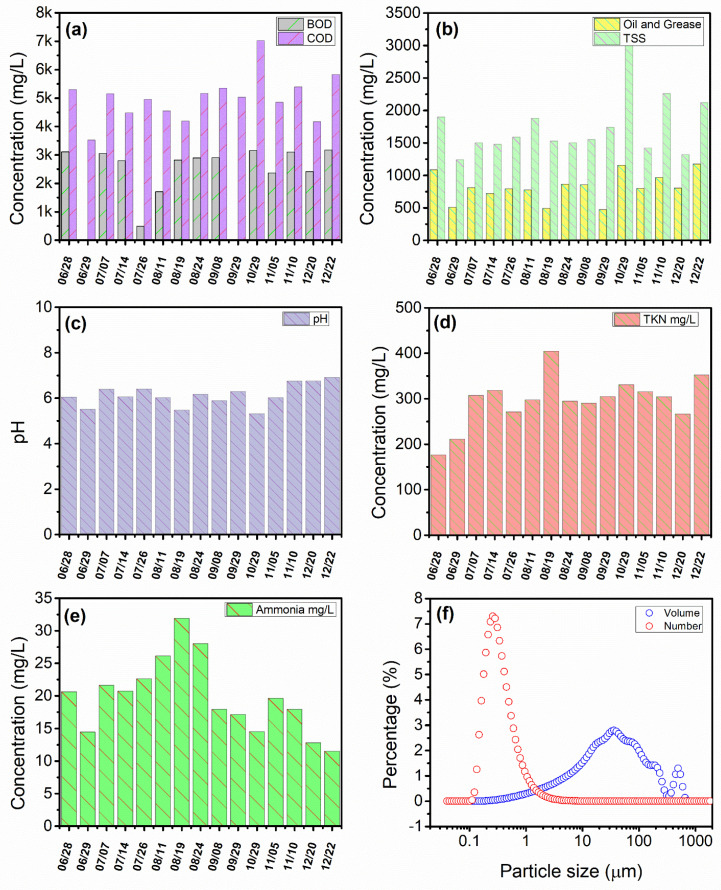
PPW characterization for different days: (**a**) BOD and COD, (**b**) oil and grease and TSS, (**c**) pH (**d**), TKN (**e**), and TSS (**f**) particle size distribution in PPW. The red circles provide the number average, and the blue circles represent the volume average particle size distribution.

PPW was taken from Tyson Food Inc. several times between June and December 2021. PPW properties varied daily, as shown in Figure 3. The wastewater collected from Tyson Food Inc. exhibited the following characteristics: an average ammonia concentration of approximately 19.8 ± 5.7 mg/L, a BOD of 2615 ± 765 mg/L, a COD of 4996 ± 811 mg/L, a pH level around 6.1, a _S_BOD of 1101 ± 178 mg/L, a TKN of 296 ± 53 mg/L, and a TSS of 1742 ± 472 mg/L (Figure 3a–e). Based on the analysis, it was concluded that the variation was independent of the day and might be due to other factors, including the quantity of chicken processed. Several reports [5,19,20] from various poultry processing facilities provided values that ranged from one-fifth to half of the values found in this investigation. But Basitere et al. [20] reported COD levels from a PPW treatment plant effluent as high as 9600 mg/L, nearly twice the average COD found in this investigation (Figure 3a).

Additionally, a study was conducted to ascertain particle size distribution in the PPW sample. All of the experiments showed that the particle size within PPW was within a constant range. PPW had particle sizes ranging from 0.05 µm to 1000 µm, with a number average of 0.28 µm and a volume average of 39.78 µm, as shown in Figure 3f. Notably, Figure 3f shows that PPW contained a larger volume of larger particles (about 39.78 µm). In contrast, the number-based average indicated that smaller particles (about 0.28 µm) were more abundant there. It is noteworthy that Sardari et al. [13] also found a comparable size distribution in their investigation of PPW from the same organization.

### 3.2. Stainless Steel Membrane Performance

#### 3.2.1. Treatment of PPW before the First DAF

**Figure 4:**
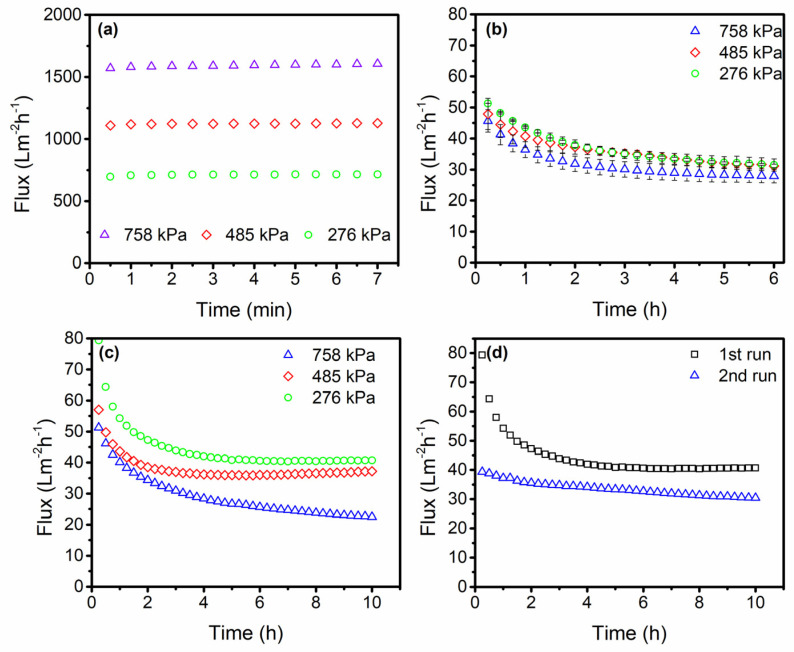
(**a**) Initial DI water flux for the 0.02 µm SSUF membrane performed at 276 kPa, 485 kPa, and 758 kPa *TMP*s for 7 min; (**b**) average of the normalized flux including the deviation for repeated primary treatment wastewater using the 0.02 µm SSUF membrane for 6 h; (**c**) normalized flux for primary treatment wastewater at different *TMP* for 10 h; (**d**) normalized flux for repeated experiments at 276 kPa *TMP* for 10 h.

In water treatment operations, SSUF membranes are currently uncommon, particularly when treating wastewater containing organic pollutants like PPW. Before employing the membrane for industrial applications, it is imperative to comprehend how SSUF performs in a lab setting. Initially, the DI water flux defines the SSUF membrane. The DI water flux is displayed at various *TMP*s in Figure 4a. According to the outcome, the flux rises linearly as *TMP* increases [5].

There are several advantages to replacing the entire DAF unit with the SSUF membrane, including lower chemical consumption and less space needed for PPW treatment. Treating PPW with 0.02 µm SSUF prior to the first DAF was the primary goal of this study. The variation in the normalized permeate flux with time is shown in Figure 4b–d. We normalized the flux to 25 °C using the viscosity at temperature (*T*) in response to the membrane’s constant temperature rise.

(4) $$Normalized\;flux=\frac{Viscosity\;of\;water\;at\left(T\right)x\;Flux\;at\;T}{Viscosity\;of\;water\;at\;25\;degree\;Celsius}$$

At a cross-flow velocity (CFV) of 1.90 m s^−1^, the SSUF performance was examined for 6 and 10 h. The two studies’ results indicate that there was a rapid drop in flux for around two hours before it gradually decreased. As seen in Figure 4b, the lowest flux was at 758 kPa and the highest permeate recovery was at 276 kPa. After two hours, the trend converged, and throughout the investigation, the behavior of the 276 kPa and 758 kPa was consistent. At 276 kPa and 483 kPa, there was a similar flux decline and permeate recovery. Two replicate tests were carried out at 758 kPa, and throughout the experiment, both displayed a similar trend. After 10 h of research on the flux behavior, the long-term behavior of the SSUF for treating PPW was understood and is shown in Figure 4d,c. Like the last study, all *TMP*s experienced a rapid flux decline in the early stages. For the 758 kPa experiment, the flux declined continuously throughout the study, but after two hours of operation, the flux stabilized at 276 kPa and 483 kPa. In line with the previously mentioned results, the result also reveals that the maximum flux was measured at 276 kPa. Based on Marchesi et al. [35], concentration polarization and fouling at higher pressures could be the cause of this. According to the previous findings, at 276 kPa, a greater volume was recovered, and the removal efficiency was similar to that of other *TMP*s at that pressure. We also conducted numerous experiments with 276 kPa, the results of which are shown in Figure 4d. Two of the results showed similar behavior and slight stability in the second run compared with the first run.

#### 3.2.2. Pathogen Removal Validation

The results of the pathogen removal by SSUF are shown in Table 2. Both PPW and the treated water were analyzed. A dilution rate of 10,000 times was used for both the coliform count and *E. coli* count for PPW, while a dilution rate of 100 times was used for the coliform count for the treated water, but there was no dilution for *E. coli* count. The SSUF successfully removed over 99% of the *E. coli* and total coliform at all of the operating *TMP*s, although the highest removal was observed at the highest *TMP*.

#### 3.2.3. Particles Removal

**Figure 5:**
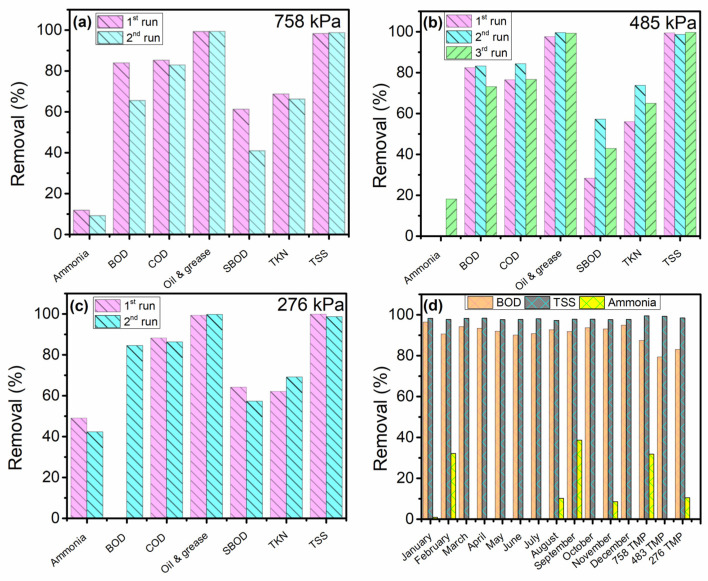
Removal efficiency of SSUF at (**a**) 758 kPa *TMP*, (**b**) 483 kPa *TMP*, (**c**) 276 kPa *TMP*, and (**d**) comparison with industrial wastewater treatment plant effluent.

**Figure 6:**
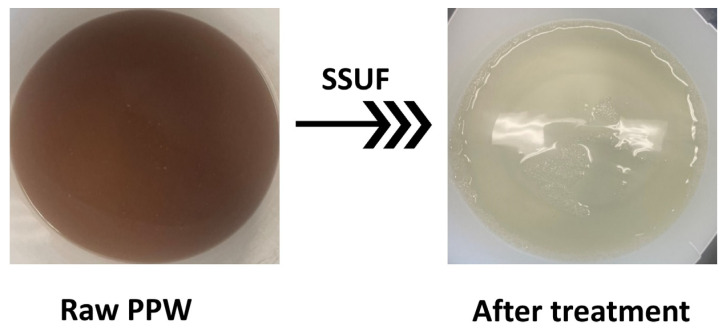
Optical images of raw PPW sample and corresponding treated water using 0.02-micron SSUF.

At the three *TMP*s, more than 90% of TSS, oil, and grease were eliminated. The percentage of particles removed by SSUF at various *TMP*s is displayed in Figure 5a–c. The TSS and oil and grease were effectively removed by SSUF. TSS and oil and grease were removed up to 100% at both 483 kPa and 758 kPa *TMP*. The amount of COD and BOD removed increased with *TMP*. The behavior of ammonia removal was different from the other parameters. There were two instances where the ammonia increased at 483 kPa *TMP*, while there was only a slight decrease in the amount of ammonia at 276 kPa and 758 kPa *TMP*. The monthly average result obtained from Tyson Food Inc.’s treatment facility was compared with the average results observed at the various *TMP*s. The information displayed the three parameters (BOD, TSS, and ammonia) that were measured from the second DAF’s effluent on a monthly average. Using Figure 5d, even after receiving two DAF treatments at the facility, the ammonia and TSS percentages that were removed from Tyson Food Inc. were comparable to the outcome from SSUF. The percentage of BOD removed, however, was much less than what was obtained in the Tyson’s facility. As illustrated in Figure 6, the permeate quality significantly improved after the treatment, and the coloring was substantially less than in the raw water. In the SSUF-treated water, the levels of oil and grease ranged from 2.9 to 12 mg/L, total TKN from 83 to 140 mg/L, and TSS from 4.5 to 20 mg/L. While the oil and grease, TKN, and TSS levels met the discharge standard, some other parameters exceeded the permissible limits. To meet discharge requirements, additional post treatment of the treated water might be needed. Our results reveal that SSUF removed 90% of the COD, aligning with a previous study involving sewage treatment with a stainless-steel membrane bioreactor [25]. In contrast, Lo et al. reported a COD value of PPW of 353.1 ± 1.1 mg/L when treating with a polymeric membrane, although their PPW underwent pretreatment, unlike the untreated PPW in our study. The initial COD concentration in their PPW was approximately 858.2 ± 2.4 mg/L, significantly lower than the initial COD concentration in our study (4996 ± 811 mg/L). These results indicate that SSUF, even without pretreatment, removed more COD (90%) compared with the polymeric membrane (59%) [17]. In a different study, a regenerated cellulose (RC) membrane with a molecular weight cutoff of 30 kDa achieved 82% COD and 87% BOD removal when treating chiller PPW. Our results show that SSUF removed a higher percentage of COD (90%). Notably, the removal efficiency improved when PPW was pretreated with electrocoagulation [13]. Despite the high initial contaminant concentration in the PPW used in our study, the performance of SSUF was comparable to other UF methods used for treating less contaminated PPW. Incorporating simple post-treatment processes with SSUF has the potential to enhance its performance further to meet the discharge standards.

**Figure 7:**
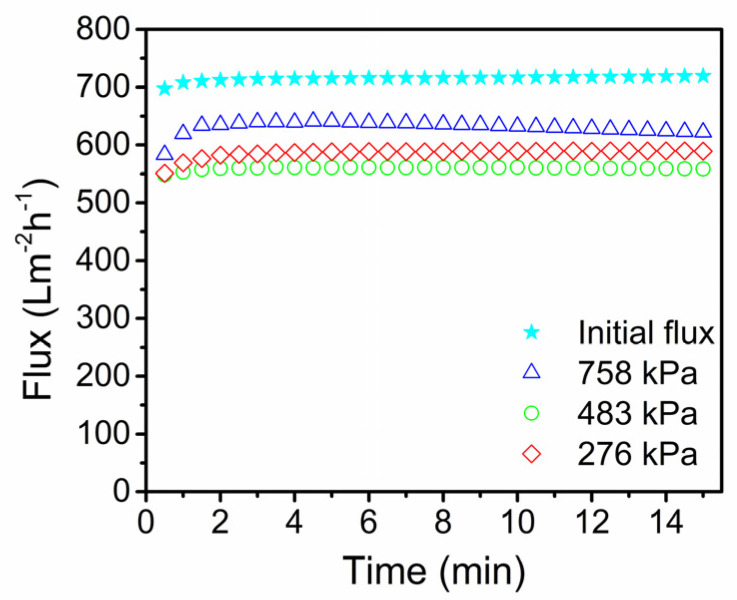
DI water flux tested at 276 kPa for the regenerated membranes after the membrane studied with PPW purification at different *TMP*s.

The membrane performance decreased drastically after each experiment due to the fouling. So, the protocol outlined in the methods section was followed to regenerate the membranes after studies at different pressures (758 kPa, 483 kPa, and 276 kPa) using Protease A, SDS, NaOH, and HNO_3_ solutions [34]. To ascertain the membrane’s recovery following regeneration, the DI water flux was measured. The plot of the regenerated membranes’ DI water flux against time following various experiments is displayed in Figure 7. With the regeneration method designed for this investigation, more than 80% of the original flux was recovered.

### 3.3. Critical Flux Evaluation

**Figure 8:**
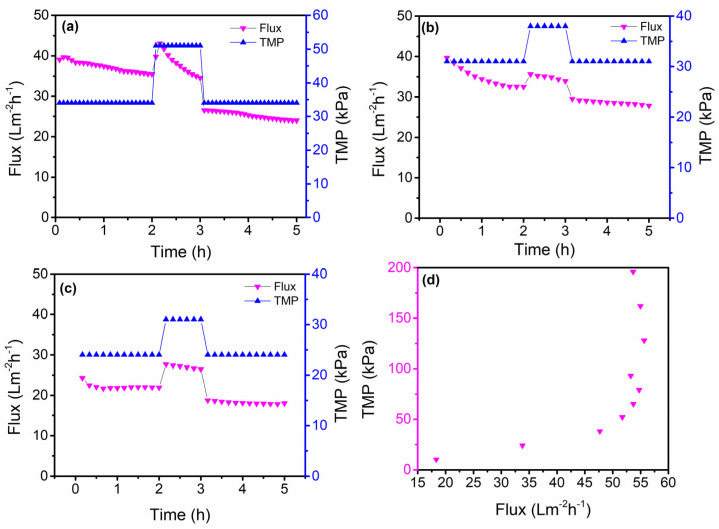
Critical flux at 1.90 m s^−1^: (**a**) plot of flux versus time at the 34 kPa and 51 kPa interchange, (**b**) plot of flux versus time at the 31 kPa and 38 kPa interchange, (**c**) plot of flux versus time at the 24 kPa and 31 kPa interchange, (**d**) and plot of *TMP* versus flux.

CFV was maintained constantly (1.90 m s^−1^) throughout the study for the critical flux measurement. All flux results were normalized to 25 °C. Firstly, we examined the pressure to which the critical flux fell, and the results of this study are shown in Figure 8a–d. The study was performed by alternating the experiment between two different *TMP*s. The first study alternated the experiment between 34 kPa and 51 kPa. As shown in Figure 8a, the flux continuously declined despite returning *TMP* to the initial value, which shows that the critical flux was below 34 kPa. In the second case, *TMP* started at 31 kPa, increased to 38 kPa, and later reduced to 31 kPa. Reducing the starting *TMP* from 34 kPa to 31 kPa showed a significant reduction in the flux decline (as shown in Figure 8b), and it was deduced that the critical flux was close to 31 kPa. The subsequent studies entailed further reducing the operating *TMP*, and the results show that the flux gradually approached a steady state as the alternating *TMP* was reduced. The results are presented in Figure 8c. This suggests that the operating *TMP* for the highly contaminated PPW was below 34 kPa. SSUF performed impressively in removing pathogens and pollutants from PPW.

We determined the critical flux by gradually increasing the *TMP*, and the corresponding flux was measured. The last point on a straight line of the flux–*TMP* relationship was assumed as the critical flux, and the corresponding pressure was the critical pressure. As shown in Figure 8d, the critical flux was about 48 Lm^−2^h^−1^ at around 38 kPa.

## 4. Conclusions

This work aimed to intensify the PPW treatment unit by replacing the current treatment method with the SSUF membrane. PPW was found to be more highly contaminated. We found that the BOD in the combined PPW could be up to five times more than the reported values in the literature. Comprehensive analyses encompassing permeate flux, pollutant removal, and pathogen elimination were conducted. Remarkably, SSUF exhibited a promising performance in PPW treatment. Even without pretreatment, a single SSUF achieved remarkable results in pathogen removal exceeding 99%, for oil and grease and TSS. Interestingly, the value of oil and grease, TKN, and TSS in the treated water met the standard for discharging the water into the environment. Although the values of COD, BOD, ammonia, and sBOD did not meet the discharge standard, SSUF showed that prior treatment or post-treatment would be sufficient for the PPW to be reusable and dischargeable. Compared with previous studies, SSUF showed better results for treating PPW without pretreatment. Even when pretreatment had been performed in some studies, SSUF showed comparable or better results [13].

Operating at 276 kPa *TMP* showed a comparable performance to higher *TMP*, indicating that reduced energy could be used to achieve the flux. Additionally, we investigated the possible ways of cleaning the membrane after filtration, with the cleaning technique demonstrating over 80% effectiveness. The overall result showed that SSUF could have a long life span because a single SSUF module was used for over a year. This study estimated the critical flux to be 48 Lm^−2^h^−1^ at a cross-flow velocity of 1.90 m s^−1^, offering valuable insights into SSUF membrane performance. Notably, mild post-treatment measures are essential for reusing the permeate to mitigate odor concerns, making SSUF an appealing alternative to conventional PPW treatment. This research represents a significant stride towards the advancement of PPW treatment methods.

## Figures and Tables

**Table 1:** Membrane specifications.

Parameters	Values
Surface area m^2^	0.0058
Length m	0.30
Pore Size (µm)	0.02
Diameter (mm)	6
Material	Stainless steel
Type	Tubular
Flow type	Tangential

**Table 2:** Pathogen removal validation using IDEXX Colilert 24–97 Well Tray (A) 276 kPa *TMP*, (B) 483 kPa *TMP*, and (C) 758 kPa *TMP* (MPN = most probable number).

Sample Name	(A) *E. coli* (MPN/100 mL) A	(A) Total Coliform (MPN/100 mL) A	(B) *E. coli* (MPN/100 mL) B	(B) Total Coliform (MPN/100 mL) B	(C) *E. coli* (MPN/100 mL) C	(C) Total Coliform (MPN/100 mL) C
PPW	>24,196,000	>24,196,000	>24,196,000	>24,196,000	>24,196,000	>24,196,000
Permeate	345	68,670	308	92,080	115	29,090
Removal %	99.999	99.7	99.999	99.6	99.999	99.9

## Data Availability

Data are contained within the article.
